# The load of hepatitis B virus reduces the immune checkpoint inhibitors efficiency in hepatocellular carcinoma patients

**DOI:** 10.3389/fimmu.2024.1480520

**Published:** 2024-11-27

**Authors:** Zhengzheng Ji, Jiasong Li, Shasha Zhang, Yuanyuan Jia, Jing Zhang, Zhanjun Guo

**Affiliations:** ^1^ Department of Rheumatology and Immunology, The Fourth Hospital of Hebei Medical University, Shijiazhuang, China; ^2^ Department of Gerontology, The Fourth Hospital of Hebei Medical University, Shijiazhuang, China

**Keywords:** hepatitis B virus, hepatitis B virus load, immune checkpoint inhibitors, hepatocellular carcinoma, antiviral therapy

## Abstract

**Introduction:**

Chronic viral infection may lead to an immunosuppressive microenvironment, whereas the association between virus-related indicators and treatment response in hepatocellular carcinoma(HCC) patients undergoing immune checkpoint inhibitors(ICIs) therapy remains a topic of debate. We aim to investigate the influence of hepatitis virus on the ICI efficiency in HCC patients through a meta-analysis.

**Methods:**

We searched PubMed, Cochrane Library, Embase, and Web of Science until 14 July 2024 to identify cohort studies involving ICIs treatments in HCC patients. We extracted data from the literature related to hepatitis B virus (HBV) infection, hepatitis C virus (HCV) infection, baseline HBV load, and antiviral therapy. Overall survival (OS) and progression-free survival (PFS) were considered as the primary endpoints, while objective response rate (ORR) was regarded as a secondary endpoint.

**Results:**

We included 55 cohort studies published between 2019 and 2024, involving a patient population of 7180 individuals. Summarized hazard ratio (HR) comparing HBV infection with non-HBV infection in the context of ICIs therapy revealed no significant association between HBV infection and either mortality risk or progression risk with the pooled HR for OS of 1.04(95%CI: 0.93-1.16, P=0.483) and the pooled HR for PFS of 1.07(95%CI:0.96-1.20, P=0.342). HBV infected patients with HCC may have better tumor response than non-HBV infected patients receiving ICIs with the combined relative risk(RR) for ORR was 1.94 (95%CI: 1.12-3.38, P=0.002). High baseline HBV load is associated with poor survival outcomes in patients with HCC who receive ICIs with the pooled HR for OS was 1.74 (95%CI: 1.27-2.37, P=0.001), thereby antiviral therapy has the potential to significantly enhance prognostic outcomes with the pooled HR for OS was 0.24 (95% CI: 0.14-0.42 P<0.001) and the pooled HR for PFS was 0.54 (95% CI: 0.33-0.89 P=0.014).

**Conclusion:**

In individuals with HCC who received ICIs, there was no notable link found between HBV or HCV infection and prognosis. However, HBV infection showed a connection with improved tumor response. A higher initial HBV load is linked to worse survival results in HCC patients undergoing ICIs treatment and antiviral therapy can significantly improve its prognosis.

## 1 Introduction

The latest global cancer statistics report indicates that primary liver cancer continues to be the third leading cause of cancer-related mortality worldwide, with 757,948 individuals succumbing to liver cancer in 2022 (1). Hepatocellular carcinoma (HCC) is the most common type of liver cancer accounting for about 75%-85% of liver cancer (1). Worldwide, the main causes of HCC remain chronic hepatitis B virus (HBV) infection, hepatitis C virus (HCV) infection and alcohol abuse, with a predominance of HBV in China, HCV in Japan, non-alcoholic fatty liver disease(NAFLD) and non-alcoholic Steatohepatitis(NASH) and alcohol in Europe and North America (2). The global incidence of HBV-related malignancies has declined since the 2000s because of the implementation of neonatal HBV vaccination programmes (3). Although the prevalence of HBV-driven HCC has declined, the incidence of NAFLD and NASH-related HCC continues to increase because of the increasing prevalence of obesity and the metabolic syndrome, which has hindered the decline of HCC incidence (4).

Due to the lack of typical clinical symptoms in patients with HCC in the early stage, most HCC is diagnosed at an advanced stage and requires systemic treatment. Sorafenib, which was approved as a first-line treatment for HCC in 2007, has improved the survival prognosis of HCC to some extent, but the median overall survival (OS) is only 10.7 months, which is far from clinical expectations (5). The results of the IMbrave150 trial are a milestone in the treatment of HCC, as atezolizumab combined with bevacizumab (median OS: 19.2 months) is significantly better than sorafenib (median OS: 13.4 months), thus international guidelines endorsed the combination regimen as the new standard of care in front-line treatment of advanced HCC (6). With the reporting of phase III global clinical trials such as CheckMate 459 (7), CARES-310 (8) and COSMIC-312 (9), immune checkpoint inhibitors (ICIs) have further developed in the systemic treatment of HCC, significantly improving the survival prognosis of liver cancer patient. Regrettably, only about 30% of patients with HCC are able to achieve partial response (PR) or complete response (CR) using ICIs therapy, so more markers are needed to screen HCC patients who would respond to ICIs therapy to achieve precise treatment.

HCC is a prototypical inflammation-driven malignancy, and the modulation of immune surveillance within the tumor microenvironment by distinct etiologies may vary, potentially impacting the effectiveness of ICIs. In a cohort of 130 patients with HCC, the presence of NAFLD was found to be significantly associated with reduced median OS following ICIs therapy (5.4 months vs 11.0 months) (10). This may be ascribed to the activation of auto-aggressive CD8+CXCR6+PD1+T lymphocytes by ICIs, which impairs effective immune surveillance and potentially contributes to the development of HCC within the tumor microenvironment (10, 11). In a large cohort study of 1232 patients with HCC, individuals with NASH-related HCC who received treatment with lenvatinib demonstrated significantly improved survival outcomes (22.2 months versus 15.1 months). These results establish a theoretical foundation for categorizing patients with advanced HCC according to the underlying etiology.

In most HCC high-risk areas(China, Eastern Africa,Egypt, Italy, and Japan), HBV infecction and HCV infection is the predominant cause in a diverse set of HCC. Compared with other causes of HCC, the tumor microenvironment of virus-associated HCC has stronger immune inhibition than other causes (1, 12). HBV can lead to PD-1 demethylation and induce functional exhaustion of CD8+ T cells in the tumor microenvironment (TME), thereby facilitating immune evasion by tumor cells (13). HBV can also induce an immunosuppressive microenvironment in the TME by influencing the polarization of tumor-associated macrophages (TAM), as well as modulating levels of IL-6 and IL-8 (14–16). Due to the intricate nature of theTME in HBV-HCC, the association between this environment and the efficacy of ICIs therapy remains a subject of intense debate. A meta-analysis of three large randomized controlled phase III clinical trials (CheckMate-459 (7), IMbrave150 (6), and KEYNOTE-240 (17)) which evaluate the clinical beneifit by comparing the ICI treatment with non-ICIs therapy treatments (including placebo and sorafenib) in advanced HCC patients, found that the difference of treatment efficiency (OS) was more remarkable for the ICI vs non-ICI analysis in HBV-HCC patients when compared with those of ICI vs non-ICI analysis in non-HBV-HCC patients, while none performed the direct comparison for the stratified analysis of ICI treatment efficency between HBV-HCC and non-HBV-HCC patients. In this article, we conduct a meta-analysis to explore the association between HBV infection and the outcomes of HCC patients undergoing ICIs therapy.

A high baseline HBV load is associated with poor prognosis in patients treated with sorafenib, as well as being an independent risk factor for low survival rates and early recurrence after curative resection in HCC patients (18–20). There is ongoing debate regarding the association between baseline HBV load and prognosis in patients with HCC undergoing treatment with ICIs. Several retrospective studies with small sample sizes have indicated that baseline viral load does not exhibit a significant correlation with OS (21), and these findings are constrained by limited sample size. We conducted a meta-analysis to further investigate the correlation between HBV load and the prognosis as well as tumor response in HCC patients treated with ICI. Previous research has indicated that antiviral therapy for HBV can significantly enhance the survival outcomes of ICIs patients undergoing antiangiogenic therapy or ICIs therapy (18, 21). Our study aims to conduct a meta-analysis to verify the impact of both HBV load and antiviral therapy on treatment efficiency of ICI in HCC patients.

## 2 Materials and methods

We conducted this meta-analysis according to the Preferred Reporting Items for Systematic Reviews and Meta-Analysis (PRISMA) guidelines.

### 2.1 Literature search strategy

We systematically searched multiple electronic databases, covering PubMed, Embase,Cochrane and Web of science for all the available articles published before 14 July 2024. The search terms mainly included the following words: “Immune checkpoint inhibitors”, “Pembrolizumab”, “Nivolumab”, “Atezolizumab”, “Durvalumab”,”Tislelizumab”, “Camrelizumab”, “Sintilimab”, “Carcinoma, Hepatocellular”, “Survival Rate”, “Prognosis”. It should be noted that only publications in English were considered for inclusion.

### 2.2 Study selection

Inclusion criteria:(1) Study design type: cohort studies about the treatment of HCC with ICIs. (2) Study object: patients diagnosed with HCC, as confirmed by imaging evidence or pathological evidence; (3) Intervention measures: ICIs monotherapy or ICIs combined with targeted drug.

Exclusion criteria: (1) Duplicated articles; (2) Articles that were reviews, Bioinformation analyses, meeting summaries, case reports, animal experiments, expert consensuses, or editorials; (3) Articles that did not specify the type of research; (4) Articles that did not provide outcomes needed; (5) Studies with too small a sample size (sample size < 40); (6) Articles in other languages than English.

### 2.3 Data extraction

Screening and data extraction processes were conducted by two independent reviewers, and the differences were resolved by a third reviewer. For each included study, the following information was extracted: name of the study, publication year, the first author, study type, geographical region, number of patients, demographics and baseline characteristics of included patients, line of therapy, treatment strategy, clinical stage, follow-up time.

### 2.4 Quality assessment.

The quality of the cohort studies was evaluated with the Newcastle Ottawa scale (NOS). There were 9 stars in the article quality evaluation, and articles with 6 stars or more were retained.

### 2.5 Statistical analysis

Stata 15.1 analysis software was used to statistically analyze the relevant outcome indicators. The summary measure was the hazard ratios (HRs) and 95% confidence interval (95%CI) for OS and progression-free survival (PFS), P < 0.05 was considered statistically significant. The Cochrane Q statistic (significant at P < 0.10) and I^2^ value (significant heterogeneity if >50%) were used to evaluate heterogeneity. If I^2^<50% or P>0.10, then the heterogeneity was considered to below and fixed-effects model was applied. Otherwise, the random-effects model was applied. The sensitivity analysis was carried out by RevMan 5.3, and the risk of publication bias was determined using Begg’s tests and Egger’s. When P > 0.05, there was considered to be no publication bias. If the number of included articles was less than 10, no further bias test was required.

## 3 Results

### 3.1 Selection process

**Figure 1:**
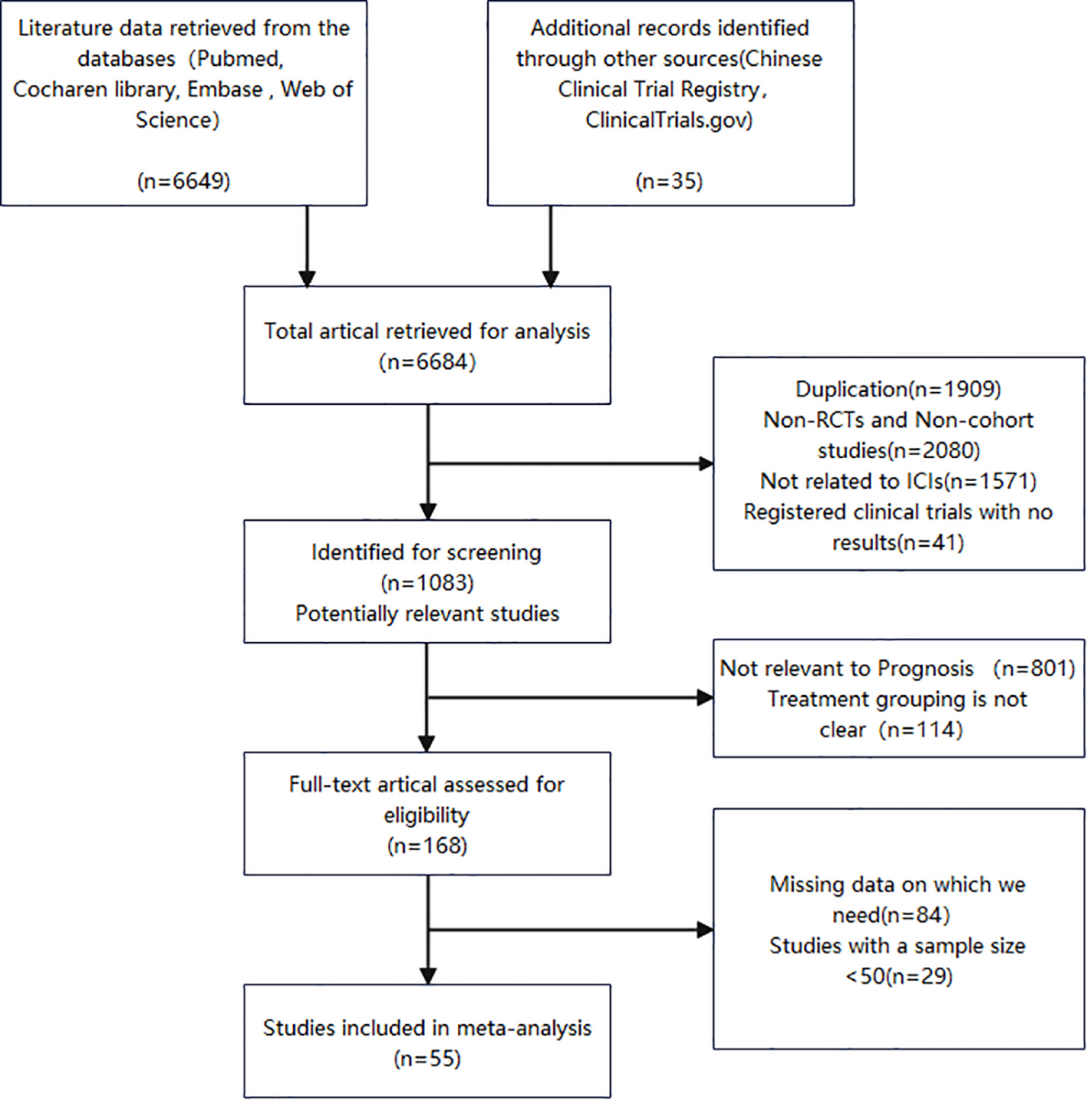
Flow diagram of the study selection.

The two reviewers independently devised search strategies. After an initial examination, a total of 6684 pertinent studies were identified, comprising 6649 records from the database search and an additional 35 records from manual searching. Among these, 1083 articles were deemed potentially relevant following title and abstract screening. Subsequent screening led to the selection of 168 articles for further evaluation. Following a thorough assessment of the remaining 168 studies’ full texts, we included 55 cohort studies published between 2019 and 2024, encompassing a patient population of 7180 individuals. **Figure 1** presents a flow chart illustrating the process employed for study selection.

### 3.2 Quality evaluation

NOS was used to evaluate the quality of the 55 cohort studies, and they were found to have a NOS score≥6, indicating medium-to-high quality(**Supplementary Table S1**).

### 3.3 Study and patient characteristics

A total of 54 enrolled articles, published between 2019 and 2024, included 54 cohort studies. Of the 54 cohort studies, 37 were from China, 5 from Taiwan China, 5 from Global, 3 form France, 1 form Korea, 1 form Austria, 1 form Thailand, 1 form USA, 1 from Singapore. In 7 studies, all patients received ICIs monotherapy; in 19 studies, patients were treated with immunotherapy combined with antiangiogenic therapy; in the other 29 studies, patients were partially treated with ICIs monotherapy and partially treated with combined immunotherapy (combined with antiangiogenic therapy or locoregional therapy). The features of the chosen studies can be observed in **Table 1**.

**Table 1:** Characteristics of the 55 cohort studies incorporated in the meta-analysis.

Study	Published	Geographical Area	Research Type	Treatment Strategy	No. of Patients total
Kennedy Yao Yi Ng et al.	2020	Singapore	RCS	ICI monotherapy/Combination ICI therapy	114
Guosheng Yuan et al.	2021	China	RCS	Combination ICI therapy	86
Haonan Liu et al.	2022	China	RCS	ICI monotherapy	54
Mengchao An et al.	2022	China	RCS	ICI monotherapy/Combination ICI therapy	217
Xuqi Sun et al.	2020	China	RCS	ICI monotherapy/Combination ICI therapy	253
Guosheng Yuan, et al.	2020	China	RCS	Combination ICI therapy	63
Rohini Sharma et al.	2021	Global	PCS	ICI monotherapy/Combination ICI therapy	420
Yanjun Shen et al.	2021	China	RCS	Combination ICI therapy	57
Shuguang Ju et al.	2022	China	RCS	Combination ICI therapy	108
Pei-Chang Lee et al.	2020	Taiwan, China	RCS	ICI monotherapy/Combination ICI therapy	90
Petros Fessas et al.	2020	Global	RCS	ICI monotherapy	233
Song Chen et al.	2021	China	RCS	Combination ICI therapy	170
Junlin Yao et al.	2021	China	RCS	Combination ICI therapy	136
Fucun Xie et al.	2022	China	RCS	Combination ICI therapy	85
Francisca-Dora Copil et al.	2023	France	RCS	Combination ICI therapy	295
Jaekyung Cheon et al.	2023	Korea	RCS	Combination ICI therapy	169
Yujing Xin et al.	2023	China	RCS	Combination ICI therapy	118
Lorenz Balcar et al.	2023	Austria	RCS	ICI monotherapy/Combination ICI therapy	72
Jing Li et al.	2023	China	RCS	Combination ICI therapy	110
Huttakan Navadurong et al.	2023	Thailand	RCS	Combination ICI therapy	83
Mathew Vithayathil et al.	2022	Global	RCS	Combination ICI therapy	191
Claudia Campani et al.	2022	France	RCS	Combination ICI therapy	75
Yue Linda Wu et al.	2022	Global	RCS	Combination ICI therapy	296
Dongbo CHEN et al.	2023	China	RCS	Combination ICI therapy	86
De-Zhen Guo et al.	2023	China	RCS	Combination ICI therapy	129
Xindan Kang et al.	2023	China	RCS	ICI monotherapy/Combination ICI therapy	85
Qingyan Liu et al.	2023	China	RCS	ICI monotherapy/Combination ICI therapy	94
Xinhua Zou et al.	2023	China	RCS	Combination ICI therapy	80
Xu Chang et al.	2023	China	RCS	Combination ICI therapy	97
Zhongjing Huang et al.	2024	China	RCS	Combination ICI therapy	123
Fei Cao et al.	2023	China	RCS	Combination ICI therapy	139
Huilan Zeng et al.	2023	China	RCS	Combination ICI therapy	152
Kang Wang et al.	2023	China	RCS	Combination ICI therapy	159
Xiaoyun Hu et al.	2022	China	RCS	Combination ICI therapy	70
Lu-shan Xiao et al.	2022	China	RCS	ICI monotherapy/Combination ICI therapy	172
Jia-Ren Wang et al.	2022	China	RCS	ICI monotherapy/Combination ICI therapy	215
Yusheng Guo et al.	2022	China	RCS	ICI monotherapy/Combination ICI therapy	97
Bang-Bin Chen et al.	2022	Taiwan, China	RCS	ICI monotherapy/Combination ICI therapy	138
Haonan Liu et al.	2022	China	RCS	ICI monotherapy	54
Bai-Bei Li et al.	2024	China	RCS	ICI monotherapy	160
Lei Xu et al.	2023	China	RCS	ICI monotherapy	85
Baizhu Xiong et al.	2023	China	RCS	ICI monotherapy	74
Jiajia Du et al.	2024	China	RCS	ICI monotherapy/Combination ICI therapy	124
Wei-Fan Hsu et al.	2023	Taiwan, China	RCS	ICI monotherapy/Combination ICI therapy	110
Lu-Shan Xiao et al.	2022	China	RCS	ICI monotherapy	161
Yue Chen et al.	2024	China	RCS	Combination ICI therapy	56
Philippe Sultanik et al.	2024	France	PCS	Combination ICI therapy	200
Jiaxin Han et al.	2024	China	RCS	ICI monotherapy/Combination ICI therapy	155
Di Pan et al.	2024	China	RCS	Combination ICI therapy	110
Darren Cowzer et al.	2024	United States	RCS	ICI monotherapy/Combination ICI therapy	91
Jiao Zhang et al.	2024	China	RCS	ICI monotherapy/Combination ICI therapy	80
Kun-Peng Ma, et al.	2024	China	RCS	Combination ICI therapy	102
Bang-Bin Chen et al.	2024	Taiwan, China	RCS	ICI monotherapy/Combination ICI therapy	143
Wen-Chi Wu et al.	2022	Taiwan, China	RCS	Combination ICI therapy	40
Michael S Lee et al.	2020	Global	PCS	ICI monotherapy/Combination ICI therapy	104

PCS, prospective cohort study; RCS, retrospective cohort study.

### 3.4 Evaluation of survival outcomes

**Figure 2:**
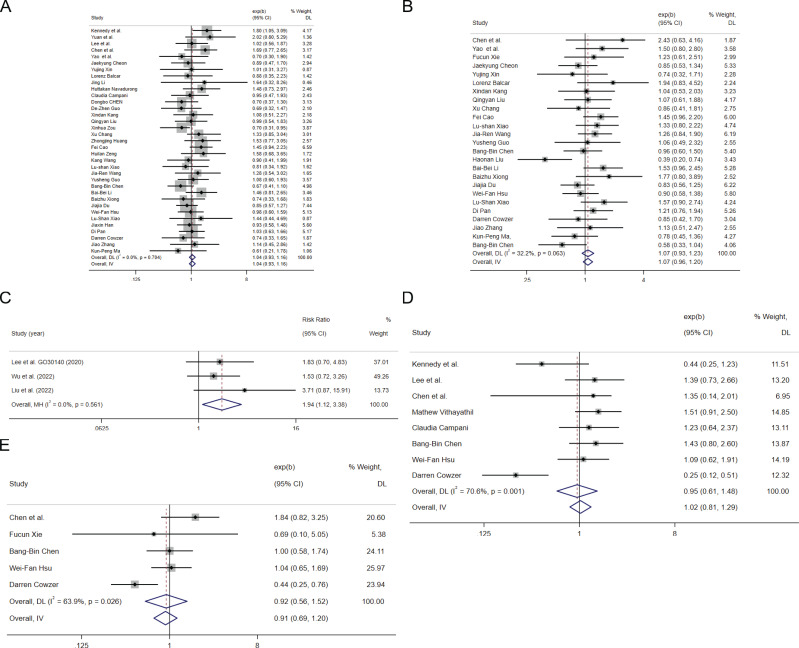
The tree diagram for HBV infection group and HCV infection group. Squares indicated study-specific effect size. Horizontal lines represent the 95% CIs. Diamonds represent the pooled effect size. The dashed vertical lines indicate the pooled effect size for immune checkpoint inhibitors. The P value for heterogeneity is obtained from the meta-analysis of the interaction. **(A)** pooled HR of OS on HBV infection vs. Non-HBV infecction; **(B)** pooled HR of PFS on HBV infection vs. Non-HBV infecction; **(C)** pooled RR of ORR for HBV infection vs. Non-HBV infecction; **(D)** pooled HR of OS on HCV infection vs. Non-HCV infecction; **(E)** pooled HR of PFS on HCV infection vs. Non-HCV infecction.

In the 35 cohort studies that provided HRs of HBV infection vs Non-HBV infection for OS (22–56), the combined HR for OS was 1.04(95%CI: 0.93-1.16, P=0.483 **Figure 2A**), indicating low heterogeneity (I^2^ = 0.0%, P=0.704), suggested that HBV infection was not associated with the risk of death in HCC patients treated with ICIs. Of the 35 cohort studies, 17 studies of 2021 HCC patients investigated the combination of ICIs with targeted therapy, whereas 3 studies evaluated ICIs monotherapy including 395 HCC patients. For the OS of HBV infection vs Non-HBV infection, subgroup analysis showed that the pooled HR of the combination of ICIs with targeted therapy group was 1.14 (95%CI: 0.96-1.35, P= 0.131, **Supplementary Figure S1A**) and that of the ICIs monotherapy group was 1.19 (95% CI:0.77-1.86, P= 0.434, **Supplementary Figure S1B**) with no significant difference between the two groups(P= 0.849).

In the 25 cohort studies that provided HRs of HBV infection vs Non-HBV infection for PFS (25–29, 35, 36, 38, 40, 57, 58), the combined HR for PFS was 1.07(95%CI:0.96-1.20, P=0.342 **Figure 2B**), indicating low heterogeneity (I^2^ = 32.2%, P=0.063), suggested that HBV infection was not associated with the risk of progression in HCC patients treated with ICIs. Of the 25 cohort studies, 13 studies of 1575 HCC patients investigated the combination of ICIs with targeted therapy and 4 studies evaluated ICIs monotherapy with 449 HCC patients. For the PFS of HBV infection vs Non-HBV infection, subgroup analysis showed that the pooled HR of the combination of ICIs with targeted therapy group was 1.12 (95%CI: 0.93-1.36, P= 0.292, **Supplementary Figure S1C**) and that of the ICIs monotherapy group was 1.1 (95% CI: 0.89-1.59, P= 0.241, **Supplementary Figure S1D**) with no significant difference between the two groups(P= 0.943).

In the 3 cohort studies that provided objective response rate (ORR) of HBV infection vs Non-HBV infection with 177 HCC patients (59–61), the combined relative risk(RR) for ORR was 1.94 (95%CI: 1.12-3.38, P=0.002 **Figure 2C**), indicating low heterogeneity (I^2^ = 0.0%, P=0.561), suggested that HBV infected patients with HCC may have better response than non-HBV infected patients receiving ICIs.

In the 7 cohort studies that provided HRs of HCV infection vs Non-HCV infection for OS (22, 24, 25, 32, 46, 50, 62), the combined HR for OS was 1.20(95%CI:0.94-1.53, P=0.236 **Figure 2D**), indicating low heterogeneity (I^2^ = 20.9%, P=0.270), suggested that HBV infection was not associated with the risk of death in HCC patients treated with ICIs. In the 4 cohort studies that provided HRs of HCV infection vs Non-HCV infection for PFS (25, 46, 50, 57), the combined HR for PFS was 1.15(95%CI:0.84-1.57, P=0.393 **Figure 2E**), indicating low heterogeneity (I^2^ = 0.0%, P=0.484), suggested that HCV infection was not associated with the risk of progression in HCC patients treated with ICIs.

**Figure 3:**
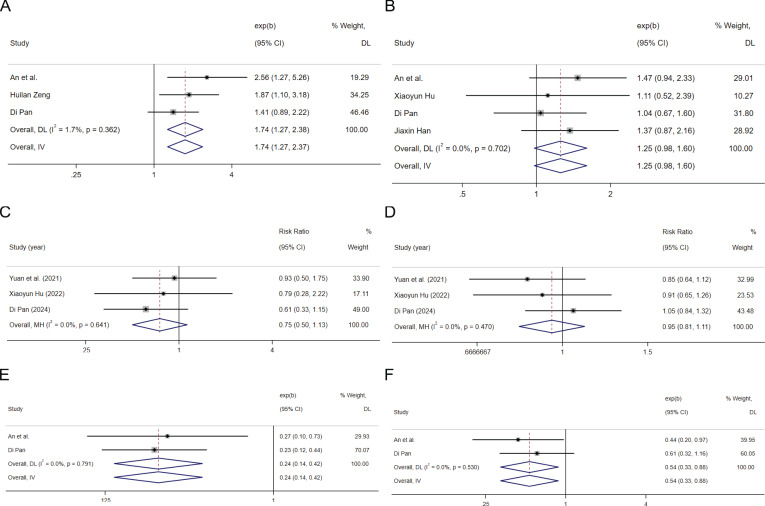
The tree diagram for baseline HBV load group and antiviral therapy group. Squares indicated study-specific effect size. Horizontal lines represent the 95% CIs. Diamonds represent the pooled effect size. The dashed vertical lines indicate the pooled effect size for immune checkpoint inhibitors. The P value for heterogeneity is obtained from the meta-analysis of the interaction. **(A)** pooled HR of OS on high HBV load vs. low HBV load; **(B)** pooled HR of PFS on high HBV load vs. low HBV load; **(C)** pooled RR of ORR for high HBV load vs. low HBV load; **(D)** pooled RR of DCR for high HBV load vs. low HBV load; **(E)** pooled HR of OS on antiviral therapy vs. Non-antiviral therapy; **(F)** pooled HR of PFS on antiviral therapy vs. Non-antiviral therapy.

In the 3 cohort studies that provided HRs of high HBV-DNA replication vs low HBV-DNA replication for OS (41, 53, 63), the combined HR for OS was 1.74(95%CI: 1.27-2.37, P=0.001 **Figure 3A**), indicating low heterogeneity (I^2^ = 1.7%, P=0.362), suggested that high HBV-DNA replication is associated with a higher risk of death in HCC patients treated with ICIs. In the 4 cohort studies that provided HRs of high HBV-DNA replication vs low HBV-DNA replication for PFS (52, 53, 63, 64), the combined HR for PFS was 1.25 (95%CI: 0.98-1.60, P=0.07 **Figure 3B**), indicating low heterogeneity (I^2^ = 0.0%, P=0.702), suggest that higher HBV-DNA replication levels tend to be associated with a higher risk of progression in HCC receiving ICIs. Three cohort studies that provided high HBV-DNA versus low HBV-DNA efficacy indicators were pooled, with an RR for ORR (53, 64, 65) of 0.75 (95%CI: 0.50-1.13, P=0.169 **Figure 3C**), and an RR for disease control rate (DCR) (53, 64, 65) of 0.95 (95%CI: 0.81-1.11, P=0.528 **Figure 3D**), suggesting that the level of HBV-DNA replication is not significantly related to treatment renponse of ICIs in patients with HCC.

Summarizing 2 articles containing data on the HR for OS and PFS in relation to receiving anti-HBV treatment versus not receiving anti-HBV treatment (53, 63), the results indicate that the HR for overall survival OS is 0.24 (95%CI: 0.14-0.42 P<0.001 **Figure 3E**), and the HR for PFS is 0.54 (95%CI: 0.33-0.89 P=0.014 **Figure 3F**). These findings suggest that administering antiviral treatment during ICIs therapy can significantly ameliorate the prognosis of patients with HCC.

Fourteen cohort studies of 2277 patients with 514 cases of alcohol-related HCC was used to evaluate the association for alcohol etiology with survival (22, 26, 31, 32, 43, 44, 50, 51, 53, 57, 66–69). The pooled HR for OS is 1.00 (95%CI: 0.84-1.20, P= 0.855 **Supplementary Figure S2A**), and the HR for PFS is 1.05 (95%CI: 0.87-1.28, P= 0.589 **Supplementary Figure S2B**). These findings indicated that alcohol etiology is not significantly associated with the prognosis of HCC patients with ICIs treatment.

There are 5 cohort studies of 772 HCC patients with 224 cases of NASH-related HCC was used to evauate the association for NASH with survival (22, 29, 31, 32, 68). The pooled HR for OS is 1.19 (95%CI: 0.92- 1.52, P= 0.181 **Supplementary Figure S2C**), suggested NASH is not significantly associated with the OS of HCC patients receiving ICIs. Only one study focus on the PFS of NASH-HCC, which made the PFS analysis uneffectively be carried out.

There are 28 cohort studies that provide HR for cirrhosis (21, 25, 26, 58, 63, 66, 70–73). The pooled HR for OS is 1.16 (95%CI: 1.04-1.31, P=0.011 **Supplementary Figure S2D**), and the HR for PFS is 1.06 (95%CI: 0.96-1.18, P=0.252 **Supplementary Figure S2E**). These findings suggest an increased risk of mortality in patients with cirrhosis for HCC patients receiving ICIs.

### 3.5 Sensitivity analysis and publication bias

**Figure 4:**
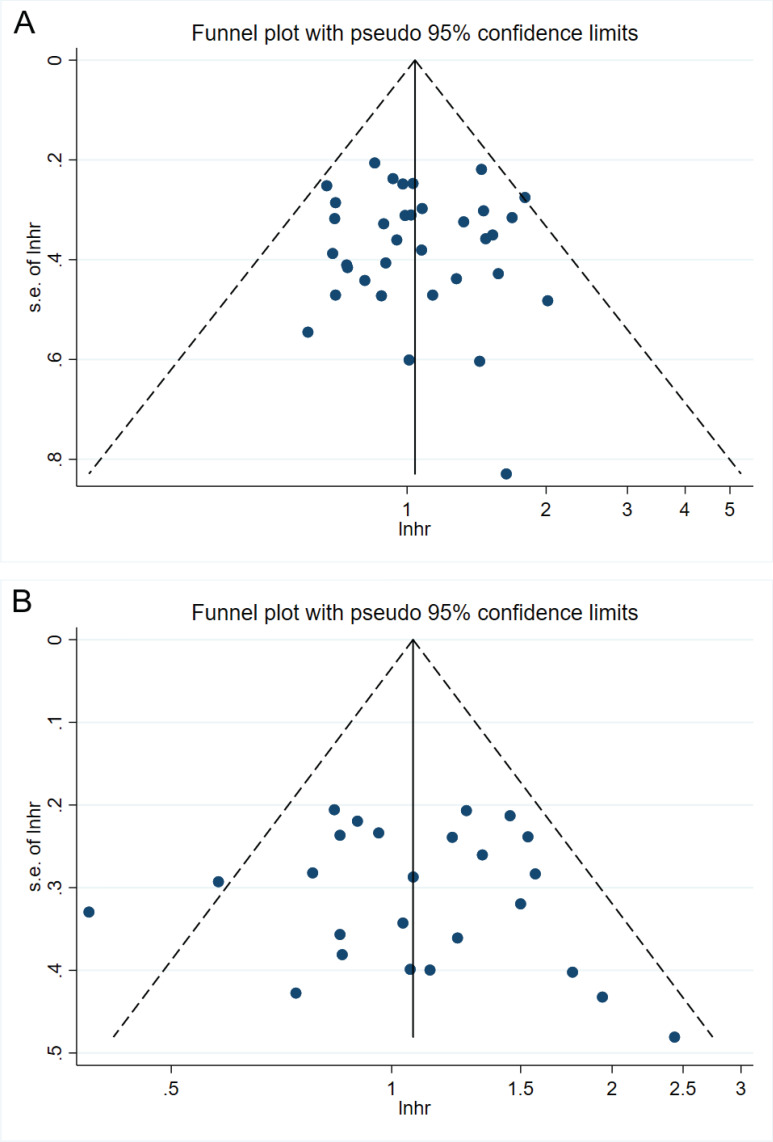
Funnel plots. **(A)** 35 cohort studies that provided HRs for OS on HBV infection vs. Non-HBV infecction. **(B)** 25 cohort studies that provided HRs for PFS on HBV infection vs. Non-HBV infecction.

To evaluate the robustness and reliability of the calculated results, a sensitivity analysis was performed(**Supplementary Figure S3**). The findings suggest that excluding any literature in this study has no impact on the obtained results (**Figure 4**, **Supplementary Figure S4**). Based on the outcomes of Begg’s tests and Egger’s tests, there is no indication of publication bias in this study(**Supplementary Table S2**).

## 4 Discussion

ICIs enhances the anti-tumor activity of the immune system by inhibiting immune downregulating factors such as programmed cell death receptor 1 (PD-1), programmed cell death ligand 1 (PD-L1), and cytotoxic T lymphocyte antigen 4 (CTLA-4) (74). ICIs exert their effects on the immune microenvironment surrounding tumors, and it has been demonstrated that CD8+ T lymphocytes, B cells, IL-6, and tertiary lymphoid structures (TLS) within tumor tissue all play a role in influencing the prognosis of ICI therapy for tumors (75–77). It has been reported that factors such as gut microbiota, antibiotic application, growth hormone, systemic inflammation response index and sarcopenia can predict the prognosis of malignant tumor patients treated with ICIs (78–84). Heterogeneity in the tumor microenvironment, influenced by various etiologies of HCC, may impact the efficacy of ICIs. Nevertheless, the association between the etiology of HCC and the prognosis as well as tumor response in patients treated with ICIs remains poorly understood. In a retrospective cohort study of 429 patients undergoing ICIs for HCC, Brown et al. observed that the three common causes of NASH, alcohol consumption, and viral infection did not exhibit a significant association with patient OS (68). Our study provides a comprehensive evaluation of the correlation between virus-related indicators and the outcomes of ICIs in patients with HCC, expanding upon the groundwork laid by our predecessors.

Our study revealed that neither HBV infection nor HCV infection exhibited a significant association with the risk of mortality or disease progression in HCC patients undergoing treatment with ICIs, and subgroup analysis also showed the HBV infection was associated with neither PFS nor OS in each subgroup (ICI monotherapy or ICI combination therapy). However, HBV infection displayed superior tumor response of HCC patients treated with ICIs compared to non-HBV infection. In a fundamental clinical trial conducted by Hsu et al, it was observed that the expression of PD-1 on tumor-infiltrating lymphocytes exhibited a statistically significant increase in patients with HBV-HCC (85). Gao et al. observed a relatively high mutation frequency of AXIN1, TSC2, ATRX, and KMT2C genes in the HBV infection cohort, which are associated with enhanced efficacy of ICIs (86). Both of these findings suggest that HBV-related HCC may benefit from ICIs therapy, however, our study only found an advantage in tumor response, without finding a significant survival benefit for HBV-infected patients receiving ICIs compared to non-HBV-infected patients. Ping-Ning Hsu et al. also found that the expression of PD-1 on tumor-infiltrating lymphocytes was lower in patients with portal vein tumor thrombosis(PVTT) compared to those without PVTT (85). In patients diagnosed with HCC, PVTT is present in 10-40% of cases (87). This provides us with a research direction for further in-depth exploration: investigating the association between PVTT or different metastasis sites in HBV-HCC patients and the outcomes following the application of ICIs. The study involving 45 HBV-related HCC patients indicate that patients with HBV-HCC harboring the HBV Pre-S2 Mutant exhibit elevated PD-L1 expression compared to other HBV-HCC patients. This observation prompts further investigation into the differential response to ICIs in HCC patients with distinct HBV mutation sites.

The viral load of other virus-associated malignancies has also been documented to impact the clinical outcomes of ICIs, including gastric cancer and anal cancer (88, 89). For HBV-HCC, multiple retrospective studies have indicated that there is no significant association between HBV load and the prognosis of ICIs therapy (19). Our meta-analysis revealed a different result, demonstrating that high HBV DNA load was correlated with an elevated risk of mortality in HCC patients undergoing ICIs treatment. In comparison with other studies, our study stands out due to its larger sample size with 6 studies and 704 patients. Firstly, HBV promotes hepatic fibrosis by integrating genes into liver cells, regulating microRNAs, promoting oxidative stress, and activating carcinogenic signaling pathways to facilitate the development of HCC. In the presence of a high HBV viral load, immune checkpoint inhibitors are unable to fully counteract the carcinogenic effects of HBV (90–93). Secondly, high HBV load itself increases the aggressiveness and metastatic potential of HCC, thus interfering with the anti-tumor effects of ICIs (93). Thirdly, a high HBV load may also lead to the upregulation of IL-6 levels through the NF-κB pathway, and elevated IL-6 has been linked to unfavorable prognosis in patients undergoing ICIs treatment (94). The aforementioned mechanisms partially account for the heightened mortality risk observed in HCC patients with elevated HBV load undergoing treatment with ICIs.

In previous studies, antiviral therapy has demonstrated efficacy in the prevention of HCC in patients with chronic hepatitis B, as well as in reducing the recurrence of HBV-related HCC and improving postoperative survival rates (95). Consistent with the retrospective study by An et al. (63), our pooled data suggest that antiviral therapy can significantly improve survival outcomes in HCC patients receiving ICIs. Effective antiviral therapy can effectively inhibit HBV replication, decrease serum viral load, expedite the seroconversion of hepatitis Be antigen (HBeAg), thereby partially alleviating or delaying liver function deterioration and enhancing survival rate (96). Antiviral therapy can also alter T cell function, with CD8+ tumor-infiltrating lymphocytes expressing higher effector T cell markers and lower T cell exhaustion markers in patients receiving antiviral therapy, playing a adjuvant role in the anti-tumor effects of ICIs (63).

Our findings have significant clinical implications, and monitoring viral load throughout HBV-HCC treatment is imperative. Timely adjustment of effective antiviral medications upon detection of viral replication can substantially impact the prognosis of patients. While antiviral therapy may enhance the clinical prognosis of HCC patients, the survival outcomes for advanced HCC patients treated with ICIs remain suboptimal. It is still necessary for researchers to actively explore predictive factors of ICIs, create prediction models, so as to precisely select the beneficiaries of immune checkpoint inhibitors and achieve individualized treatment.

The previous report including 130 HCC patients with 13 cases of NASH-related HCC demonstrated that NASH was associated with a poorer prognosis for the HCC patients with ICIs treatment (10). But the association for NASH with the poorer prognosis of ICI treatment was not found in our analysis which including 772 HCC patients with 224 cases of NASH-related HCC. The accuracy of the final results may be affected by the research subjects from different regions and ethnicities, the different etiologies of HCC included in control groups, the different ICI antibodies and the different follow-up periods.

There are some limitations to this study. Firstly, some of the included studies were retrospective cohort studies with limitations and inevitable selection bias. Secondly, although the review was not officially recorded, we conducted the meta-analysis adhering strictly to the guidelines outlined in the PRISMA statement. Furthermore, the fact that this meta-analysis did not explore the impact for HBV mutation sites, portal vein thrombosis, or different metastasis sites on the prognosis of HBV-HCC patients with ICIs treatment might affect the accuracy of the analysis. Although we previously identified outcome associated mutations for postoperative HCC patients and furtherly performed the functional analysis for their contribution on HCC growth, the ICI treatment efficiency analysis requires a large number of patients as the frequency for each candidate mutation does not exceed 20% (97, 98). Further subgroup analyses should be conducted according to HBV mutation sites, portal vein thrombus, etc. to further assess the relationship between these factors and the prognosis of HBV-HCC patients treated with ICIs.

## 5 Conclusion

In patients with HCC treated with ICIs, there was no significant correlation between HBV or HCV infection with prognosis, while HBV infection was associated with better tumor response. Higher baseline HBV load is associated with poorer survival outcomes in patients with HCC who receive ICIs therapy, and antiviral therapy can significantly improve the prognosis.

## Data Availability

The original contributions presented in the study are included in the article/**Supplementary Material**. Further inquiries can be directed to the corresponding author/s.

## References

[1] Bray F Laversanne M Sung H Ferlay J Siegel RL Soerjomataram I . Global cancer statistics 2022: GLOBOCAN estimates of incidence and mortality worldwide for 36 cancers in 185 countries. CA: Cancer J Clin. (2024) 74:229–63. doi: 10.3322/caac.21834 38572751

[2] Pinto E Meneghel P Farinati F Russo FP Pelizzaro F Gambato M . Efficacy of immunotherapy in hepatocellular carcinoma: Does liver disease etiology have a role? Digestive Liver Dis: Off J Ital Soc Gastroenterol Ital Assoc Study Liver. (2024) 56:579–88. doi: 10.1016/j.dld.2023.08.062 37758610

[3] Forner A Reig M Bruix J . Hepatocellular carcinoma. Lancet (London England). (2018) 391:1301–14. doi: 10.1016/s0140-6736(18)30010-2 29307467

[4] Gawrieh S Dakhoul L Miller E Scanga A deLemos A Kettler C . Characteristics, aetiologies and trends of hepatocellular carcinoma in patients without cirrhosis: a United States multicentre study. Alimentary Pharmacol Ther. (2019) 50:809–21. doi: 10.1111/apt.15464 31475372

[5] Llovet JM Ricci S Mazzaferro V Hilgard P Gane E Blanc JF . Sorafenib in advanced hepatocellular carcinoma. New Engl J Med. (2008) 359:378–90. doi: 10.1056/NEJMoa0708857 18650514

[6] Cheng AL Qin S Ikeda M Galle PR Ducreux M Kim TY . Updated efficacy and safety data from IMbrave150: Atezolizumab plus bevacizumab vs. sorafenib for unresectable hepatocellular carcinoma. J Hepatol. (2022) 76:862–73. doi: 10.1016/j.jhep.2021.11.030 34902530

[7] Yau T Park JW Finn RS Cheng AL Mathurin P Edeline J . Nivolumab versus sorafenib in advanced hepatocellular carcinoma (CheckMate 459): a randomised, multicentre, open-label, phase 3 trial. Lancet Oncol. (2022) 23:77–90. doi: 10.1016/s1470-2045(21)00604-5 34914889

[8] Qin S Chan SL Gu S Bai Y Ren Z Lin X . Camrelizumab plus rivoceranib versus sorafenib as first-line therapy for unresectable hepatocellular carcinoma (CARES-310): a randomised, open-label, international phase 3 study. Lancet (London England). (2023) 402:1133–46. doi: 10.1016/s0140-6736(23)00961-3 37499670

[9] Kelley RK Rimassa L Cheng AL Kaseb A Qin S Zhu AX . Cabozantinib plus atezolizumab versus sorafenib for advanced hepatocellular carcinoma (COSMIC-312): a multicentre, open-label, randomised, phase 3 trial. Lancet Oncol. (2022) 23:995–1008. doi: 10.1016/s1470-2045(22)00326-6 35798016

[10] Pfister D Núñez NG Pinyol R Govaere O Pinter M Szydlowska M . NASH limits anti-tumour surveillance in immunotherapy-treated HCC. Nature. (2021) 592:450–6. doi: 10.1038/s41586-021-03362-0 PMC8046670 33762733

[11] Yahoo N Dudek M Knolle P Heikenwälder M . Role of immune responses in the development of NAFLD-associated liver cancer and prospects for therapeutic modulation. J Hepatol. (2023) 79:538–51. doi: 10.1016/j.jhep.2023.02.033 36893854

[12] Liu X Li M Wang X Dang Z Jiang Y Wang X . PD-1(+) TIGIT(+) CD8(+) T cells are associated with pathogenesis and progression of patients with hepatitis B virus-related hepatocellular carcinoma. Cancer Immunol Immunother: CII. (2019) 68:2041–54. doi: 10.1007/s00262-019-02426-5 PMC11028102 31720814

[13] Bosch M Kallin N Donakonda S Zhang JD Wintersteller H Hegenbarth S . A liver immune rheostat regulates CD8 T cell immunity in chronic HBV infection. Nature. (2024) 631:867–75. doi: 10.1038/s41586-024-07630-7 PMC11269190 38987588

[14] Fei Y Wang Z Huang M Wu X Hu F Zhu J . MiR-155 regulates M2 polarization of hepatitis B virus-infected tumour-associated macrophages which in turn regulates the Malignant progression of hepatocellular carcinoma. J Viral Hepatitis. (2023) 30:417–26. doi: 10.1111/jvh.13809 36704832

[15] Xia C Zhu W Huang C Lou G Ye B Chen F . Genetic polymorphisms of interleukin-6 influence the development of hepatitis B virus-related liver cirrhosis in the Han Chinese population. Infection Genet Evolution: J Mol Epidemiol Evolutionary Genet Infect Dis. (2020) 84:104331. doi: 10.1016/j.meegid.2020.104331 32353512

[16] Tsuge M Hiraga N Zhang Y Yamashita M Sato O Oka N . Endoplasmic reticulum-mediated induction of interleukin-8 occurs by hepatitis B virus infection and contributes to suppression of interferon responsiveness in human hepatocytes. Virology. (2018) 525:48–61. doi: 10.1016/j.virol.2018.08.020 30240958

[17] Finn RS Ryoo BY Merle P Kudo M Bouattour M Lim HY . Pembrolizumab as second-line therapy in patients with advanced hepatocellular carcinoma in KEYNOTE-240: A randomized, double-blind, phase III trial. J Clin Oncol: Off J Am Soc Clin Oncol. (2020) 38:193–202. doi: 10.1200/jco.19.01307 31790344

[18] Yang Y Wen F Li J Zhang P Yan W Hao P . A high baseline HBV load and antiviral therapy affect the survival of patients with advanced HBV-related HCC treated with sorafenib. Liver International: Off J Int Assoc Study Liver. (2015) 35:2147–54. doi: 10.1111/liv.12805 25676812

[19] Sohn W Paik YH Kim JM Kwon CH Joh JW Cho JY . HBV DNA and HBsAg levels as risk predictors of early and late recurrence after curative resection of HBV-related hepatocellular carcinoma. Ann Surg Oncol. (2014) 21:2429–35. doi: 10.1245/s10434-014-3621-x 24619495

[20] Li ZL Yan WT Zhang J Zhao YJ Lau WY Mao XH . Identification of actual 10-year survival after hepatectomy of HBV-related hepatocellular carcinoma: a multicenter study. J Gastrointestinal Surgery: Off J Soc Surg Alimentary Tract. (2019) 23:288–96. doi: 10.1007/s11605-018-4006-4 30334177

[21] Sun X Hu D Yang Z Liu Z Wang J Chen J . Baseline HBV loads do not affect the prognosis of patients with hepatocellular carcinoma receiving anti-programmed cell death-1 immunotherapy. J Hepatocellular Carcinoma. (2020) 7:337–45. doi: 10.2147/jhc.S278527 PMC7718972 33294424

[22] Ng KYY Wong LWJ Ang AJS Tan SH Choo SP Tai DW . Real-world efficacy and safety of immune checkpoint inhibitors in advanced hepatocellular carcinoma: Experience of a tertiary Asian Center. Asia Pac J Clin Oncol. (2021) 17:e249–61. doi: 10.1111/ajco.13454 32875742

[23] Yuan G Cheng X Li Q Zang M Huang W Fan W . Safety and efficacy of camrelizumab combined with apatinib for advanced hepatocellular carcinoma with portal vein tumor thrombus: A multicenter retrospective study. OncoTargets Ther. (2020) 13:12683–93. doi: 10.2147/ott.S286169 PMC7734076 33328740

[24] Lee PC Chao Y Chen MH Lan KH Lee CJ Lee IC . Predictors of response and survival in immune checkpoint inhibitor-treated unresectable hepatocellular carcinoma. Cancers. (2020) 12:182. doi: 10.3390/cancers12010182 31940757 PMC7017111

[25] Chen S Xu B Wu Z Wang P Yu W Liu Z . Pembrolizumab plus lenvatinib with or without hepatic arterial infusion chemotherapy in selected populations of patients with treatment-naive unresectable hepatocellular carcinoma exhibiting PD-L1 staining: a multicenter retrospective study. BMC Cancer. (2021) 21:1126. doi: 10.1186/s12885-021-08858-6 34670506 PMC8527794

[26] Yao J Zhu X Wu Z Wei Q Cai Y Zheng Y . Efficacy and safety of PD-1 inhibitor combined with antiangiogenic therapy for unresectable hepatocellular carcinoma: A multicenter retrospective study. Cancer Med. (2022) 11:3612–22. doi: 10.1002/cam4.4747 PMC9554456 35403359

[27] Cheon J Kim H Kim HS Kim CG Kim I Kang B . Atezolizumab plus bevacizumab in patients with child-Pugh B advanced hepatocellular carcinoma. Ther Adv Med Oncol. (2023) 15:1–11. doi: 10.1177/17588359221148541 PMC10495918 37705533

[28] Xin Y Zhang X Liu N Peng G Huang X Cao X . Efficacy and safety of lenvatinib plus PD-1 inhibitor with or without transarterial chemoembolization in unresectable hepatocellular carcinoma. Hepatol Int. (2023) 17:753–64. doi: 10.1007/s12072-023-10502-3 37038024

[29] Balcar L Bauer D Pomej K Meischl T Mandorfer M Reiberger T . Early changes in immunoglobulin G levels during immune checkpoint inhibitor treatment are associated with survival in hepatocellular carcinoma patients. PloS One. (2023) 18:e0282680. doi: 10.1371/journal.pone.0282680 37027398 PMC10081755

[30] Kaneko S Asahina Y Murakawa M Ueyama S Maeyashiki C Watanabe H . Prognostic significance of C-reactive protein in unresectable hepatocellular carcinoma treated with atezolizumab and bevacizumab. Hepatol Res: Off J Japan Soc Hepatol. (2024) 54:562–74. doi: 10.1111/hepr.14001 38133587

[31] Navadurong H Prasoppokakorn T Siriwong N Phathong C Teeyapun N Tanasanvimon S . Modified albumin-bilirubin predicted survival of unresectable hepatocellular carcinoma patients treated with immunotherapy. World J Gastrointestinal Oncol. (2023) 15:1771–83. doi: 10.4251/wjgo.v15.i10.1771 PMC10631433 37969413

[32] Campani C Bamba-Funck J Campion B Sidali S Blaise L Ganne-Carrié N . Baseline ALBI score and early variation of serum AFP predicts outcomes in patients with HCC treated by atezolizumab-bevacizumab. Liver International: Off J Int Assoc Study Liver. (2023) 43:708–17. doi: 10.1111/liv.15487 36444741

[33] Chen D Chen X Xu L Wang Y Zhu L Kang M . Camrelizumab combined with apatinib in the treatment of patients with hepatocellular carcinoma: a real-world assessment. Neoplasma. (2023) 70:580–7. doi: 10.4149/neo_2023_230413N206 37789782

[34] Guo DZ Zhang SY Dong SY Yan JY Wang YP Cao Y . Circulating immune index predicting the prognosis of patients with hepatocellular carcinoma treated with lenvatinib and immunotherapy. Front Oncol. (2023) 13:1109742. doi: 10.3389/fonc.2023.1109742 36910622 PMC9997675

[35] Kang X Wang J Kang X Bai L . Predictive value of prognostic nutritional index (PNI) in recurrent or unresectable hepatocellular carcinoma received anti-PD1 therapy. BMC Cancer. (2023) 23:787. doi: 10.1186/s12885-023-11166-w 37612634 PMC10463676

[36] Liu Q Li R Li L Wang G Ji S Zheng X . Efficacy and safety of anti-PD-1 monotherapy versus anti-PD-1 antibodies plus lenvatinib in patients with advanced hepatocellular carcinoma: a real-world experience. Ther Adv Med Oncol. (2023) 15:1–15. doi: 10.1177/17588359231206274 PMC10599113 37885459

[37] Zou X Xu Q You R Yin G . Evaluating the benefits of TACE combined with lenvatinib plus PD-1 inhibitor for hepatocellular carcinoma with portal vein tumor thrombus. Adv Ther. (2023) 40:1686–704. doi: 10.1007/s12325-023-02449-6 36805422

[38] Chang X Wu H Ning S Li X Xie Y Shao W . Hepatic arterial infusion chemotherapy combined with lenvatinib plus humanized programmed death receptor-1 in patients with high-risk advanced hepatocellular carcinoma: A real-world study. J Hepatocellular Carcinoma. (2023) 10:1497–509. doi: 10.2147/jhc.S418387 PMC10493101 37701565

[39] Huang Z Wu Z Zhang L Yan L Jiang H Ai J . The safety and efficacy of TACE combined with HAIC, PD-1 inhibitors, and tyrosine kinase inhibitors for unresectable hepatocellular carcinoma: a retrospective study. Front Oncol. (2024) 14:1298122. doi: 10.3389/fonc.2024.1298122 38318115 PMC10838967

[40] Cao F Shi C Zhang G Luo J Zheng J Hao W . Improved clinical outcomes in advanced hepatocellular carcinoma treated with transarterial chemoembolization plus atezolizumab and bevacizumab: a bicentric retrospective study. BMC Cancer. (2023) 23:873. doi: 10.1186/s12885-023-11389-x 37718456 PMC10506240

[41] Zeng H Zhang D Yang Z Hu Z Yang Z Fu Y . Cholesterol and C-reactive protein prognostic score predicted prognosis of immune checkpoint inhibitors based interventional therapies for intermediate-to-advanced hepatocellular carcinoma patients. Int Immunopharmacol. (2023) 115:109651. doi: 10.1016/j.intimp.2022.109651 36638663

[42] Wang K Xiang YJ Yu HM Cheng YQ Feng JK Liu ZH . Overall survival of patients with hepatocellular carcinoma treated with sintilimab and disease outcome after treatment discontinuation. BMC Cancer. (2023) 23:1017. doi: 10.1186/s12885-023-11485-y 37867191 PMC10591394

[43] Xiao LS Li RN Cui H Hong C Huang CY Li QM . Use of computed tomography-derived body composition to determine the prognosis of patients with primary liver cancer treated with immune checkpoint inhibitors: a retrospective cohort study. BMC Cancer. (2022) 22:737. doi: 10.1186/s12885-022-09823-7 35794525 PMC9258103

[44] Wang JR Li RN Huang CY Hong C Li QM Zeng L . Impact of antibiotics on the efficacy of immune checkpoint inhibitors in the treatment of primary liver cancer. Liver Res. (2022) 6:175–80. doi: 10.1016/j.livres.2022.05.004.Embase PMC11791789 39958197

[45] Guo Y Ren Y Wu F Dong X Zheng C . Prognostic impact of sarcopenia in patients with hepatocellular carcinoma treated with PD-1 inhibitor. Ther Adv Gastroenterol. (2022) 15:1–14. doi: 10.1177/17562848221142417 PMC9806410 36600683

[46] Chen BB Liang PC Shih TT Liu TH Shen YC Lu LC . Sarcopenia and myosteatosis are associated with survival in patients receiving immunotherapy for advanced hepatocellular carcinoma. Eur Radiol. (2023) 33:512–22. doi: 10.1007/s00330-022-08980-4 35864351

[47] Li BB Chen LJ Lu SL Lei B Yu GL Yu SP . C-reactive protein to albumin ratio predict responses to programmed cell death-1 inhibitors in hepatocellular carcinoma patients. World J Gastrointestinal Oncol. (2024) 16:61–78. doi: 10.4251/wjgo.v16.i1.61 PMC10824115 38292845

[48] Xiong B Fu B Wu Y Gao F Hou C . Body composition predicts prognosis of hepatocellular carcinoma patients undergoing immune checkpoint inhibitors. J Cancer Res Clin Oncol. (2023) 149:11607–17. doi: 10.1007/s00432-023-05051-z PMC11797616 37400572

[49] Du J Huang Z Zhang E . Nomograms confirm serum IL-6 and CRP as predictors of immune checkpoint inhibitor efficacy in unresectable hepatocellular carcinoma. Front Immunol. (2024) 15:1329634. doi: 10.3389/fimmu.2024.1329634 38304429 PMC10830723

[50] Hsu WF Lai HC Chen CK Wang HW Chuang PH Tsai MH . Combined CRAFITY score and α-fetoprotein response predicts treatment outcomes in patients with unresectable hepatocellular carcinoma receiving anti-programmed death-1 blockade-based immunotherapy. Am J Cancer Res. (2023) 13:654–68. PMC9989621 36895987

[51] Xiao LS Hu CY Cui H Li RN Hong C Li QM . Splenomegaly in predicting the survival of patients with advanced primary liver cancer treated with immune checkpoint inhibitors. Cancer Med. (2022) 11:4880–8. doi: 10.1002/cam4.4818 PMC9761067 35599583

[52] Han J Kuai W Yang L Tao X Wang Y Zeng M . Impact of metabolic dysfunction-associated steatotic liver disease on the efficacy of immunotherapy in patients with chronic hepatitis B-related hepatocellular carcinoma. Cancer Biol Med. (2024) 21:814–25. doi: 10.20892/j.issn.2095-3941.2024.0048 PMC11414222 38712819

[53] Pan D Liu HN Yao ZY Chen XX Li YQ Zhu JJ . Impact of baseline hepatitis B virus viral load on the long-term prognosis of advanced hepatocellular carcinoma treated with immunotherapy. World J Gastrointestinal Oncol. (2024) 16:2504–19. doi: 10.4251/wjgo.v16.i6.2504 PMC11236260 38994160

[54] Cowzer D Chou JF Walch H Keane F Khalil D Shia J . Clinicogenomic predictors of outcomes in patients with hepatocellular carcinoma treated with immunotherapy. Oncologist. (2024) 29:894–903. doi: 10.1093/oncolo/oyae110 PMC11448888 38937977

[55] Zhang J Yin Y Tang J Zhang Y Tian Y Sun F . Changes in serum interleukin-8 levels predict response to immune checkpoint inhibitors immunotherapy in unresectable hepatocellular carcinoma patients. J Inflammation Res. (2024) 17:3397–406. doi: 10.2147/jir.S460931 PMC11135337 38813541

[56] Ma KP Fu JX Duan F Wang MQ . Efficacy and predictive factors of transarterial chemoembolization combined with lenvatinib plus programmed cell death protein-1 inhibition for unresectable hepatocellular carcinoma. World J Gastrointestinal Oncol. (2024) 16:1236–47. doi: 10.4251/wjgo.v16.i4.1236 PMC11037041 38660650

[57] Xie F Chen B Yang X Wang H Zhang G Wang Y . Efficacy of immune checkpoint inhibitors plus molecular targeted agents after the progression of lenvatinib for advanced hepatocellular carcinoma. Front Immunol. (2022) 13:1052937. doi: 10.3389/fimmu.2022.1052937 36569829 PMC9780480

[58] Chen BB Liang PC Shih TT Liu TH Shen YC Lu LC . Changes in Posttreatment Spleen Volume Associated with Immunotherapy Outcomes for Advanced Hepatocellular Carcinoma. J Hepatocell Carcinoma. (2024) 11:1015–29. doi: 10.2147/jhc.S462470 PMC11162638 38854818

[59] Wu WC Lin TY Chen MH Hung YP Liu CA Lee RC . Lenvatinib combined with nivolumab in advanced hepatocellular carcinoma-real-world experience. Invest New Drugs. (2022) 40:789–97. doi: 10.1007/s10637-022-01248-0 PMC9288359 35477812

[60] Lee MS Ryoo BY Hsu CH Numata K Stein S Verret W . Atezolizumab with or without bevacizumab in unresectable hepatocellular carcinoma (GO30140): an open-label, multicentre, phase 1b study. Lancet Oncol. (2020) 21:808–20. doi: 10.1016/s1470-2045(20)30156-x 32502443

[61] Liu H Qin X Xu Z Wu M Lu T Zhou S . Comparison of effectiveness and safety of camrelizumab between HBV-related and non-B, non-C hepatocellular carcinoma: A retrospective study in China. Front Genet. (2022) 13:1000448. doi: 10.3389/fgene.2022.1000448 36160021 PMC9500546

[62] Vithayathil M D’Alessio A Fulgenzi CAM Nishida N Schönlein M von Felden J . Impact of older age in patients receiving atezolizumab and bevacizumab for hepatocellular carcinoma. Liver International: Off J Int Assoc Study Liver. (2022) 42:2538–47. doi: 10.1111/liv.15405 PMC9825835 35986902

[63] An M Wang W Zhang J Till BG Zhao L Huang H . Association of hepatitis B virus DNA levels with overall survival for advanced hepatitis B virus-related hepatocellular carcinoma under immune checkpoint inhibitor therapy. Cancer Immunol Immunother: CII. (2023) 72:385–95. doi: 10.1007/s00262-022-03254-w PMC10992824 35907016

[64] Hu X Li R Li Q Zang M Yuan G Chen J . Interaction between baseline HBV loads and the prognosis of patients with HCC receiving anti-PD-1 in combination with antiangiogenic therapy undergoing concurrent TAF prophylaxis. BMC Infect Dis. (2022) 22:614. doi: 10.1186/s12879-022-07602-0 35836207 PMC9284788

[65] Yuan G Li R Li Q Hu X Ruan J Fan W . Interaction between hepatitis B virus infection and the efficacy of camrelizumab in combination with apatinib therapy in patients with hepatocellular carcinoma: a multicenter retrospective cohort study. Ann Transl Med. (2021) 9:1412. doi: 10.21037/atm-21-3020 34733964 PMC8506751

[66] Copil FD Campani C Lequoy M Sultanik P Blaise L Wagner M . No correlation between MASLD and poor outcome of Atezolizumab-Bevacizumab therapy in patients with advanced HCC. Liver International: Off J Int Assoc Study Liver. (2024) 44:931–43. doi: 10.1111/liv.15833 38291735

[67] Xu L Chen L Zhang B Liu Z Liu Q Liang H . Alkaline phosphatase combined with γ-glutamyl transferase is an independent predictor of prognosis of hepatocellular carcinoma patients receiving programmed death-1 inhibitors. Front Immunol. (2023) 14:1115706. doi: 10.3389/fimmu.2023.1115706 36761721 PMC9905229

[68] Brown TJ Mamtani R Gimotty PA Karasic TB Yang YX . Outcomes of hepatocellular carcinoma by etiology with first-line atezolizumab and bevacizumab: a real-world analysis. J Cancer Res Clin Oncol. (2023) 149:2345–54. doi: 10.1007/s00432-023-04590-9 PMC11044531 36862158

[69] Yano Y Yamamoto A Mimura T Kushida S Hirohata S Yoon S . Factors associated with the response to atezolizumab/bevacizumab combination therapy for hepatocellular carcinoma. JGH Open: Open Access J Gastroenterol Hepatol. (2023) 7:476–81. doi: 10.1002/jgh3.12932 PMC10366485 37496817

[70] Sharma R Pillai A Marron TU Fessas P Saeed A Jun T . Patterns and outcomes of subsequent therapy after immune checkpoint inhibitor discontinuation in HCC. Hepatol Commun. (2022) 6:1776–85. doi: 10.1002/hep4.1927 PMC9234627 35481940

[71] Shen Y Wang H Wei J Li W . Early prediction of objective response of fibrinogen in a real-world cohort of hepatocellular carcinoma cases treated by programmed cell death receptor-1 and lenvatinib. OncoTargets Ther. (2021) 14:5019–26. doi: 10.2147/ott.S332351 PMC8513529 34675546

[72] Ju S Zhou C Yang C Wang C Liu J Wang Y . Apatinib plus camrelizumab with/without chemoembolization for hepatocellular carcinoma: A real-world experience of a single center. Front Oncol. (2021) 11:835889. doi: 10.3389/fonc.2021.835889 35174073 PMC8841670

[73] Fessas P Kaseb A Wang Y Saeed A Szafron D Jun T . Post-registration experience of nivolumab in advanced hepatocellular carcinoma: an international study. J Immunother Cancer. (2020) 8:e001033. doi: 10.1136/jitc-2020-001033 32868393 PMC7462152

[74] Postow MA Sidlow R Hellmann MD . Immune-related adverse events associated with immune checkpoint blockade. New Engl J Med. (2018) 378:158–68. doi: 10.1056/NEJMra1703481 29320654

[75] Zhang Y Chen H Mo H Hu X Gao R Zhao Y . Single-cell analyses reveal key immune cell subsets associated with response to PD-L1 blockade in triple-negative breast cancer. Cancer Cell. (2021) 39:1578–1593.e1578. doi: 10.1016/j.ccell.2021.09.010 34653365

[76] Myojin Y Kodama T Sakamori R Maesaka K Matsumae T Sawai Y . Interleukin-6 is a circulating prognostic biomarker for hepatocellular carcinoma patients treated with combined immunotherapy. Cancers. (2022) 14:883. doi: 10.3390/cancers14040883 35205631 PMC8870238

[77] Cascone T Kar G Spicer JD García-Campelo R Weder W Daniel DB . Neoadjuvant durvalumab alone or combined with novel immuno-oncology agents in resectable lung cancer: the phase II neoCOAST platform trial. Cancer Discovery. (2023) 13:2394–411. doi: 10.1158/2159-8290.Cd-23-0436 PMC10618740 37707791

[78] Zhao M Duan X Han X Wang J Han G Mi L . Sarcopenia and systemic inflammation response index predict response to systemic therapy for hepatocellular carcinoma and are associated with immune cells. Front Oncol. (2022) 12:854096. doi: 10.3389/fonc.2022.854096 35463384 PMC9024177

[79] Yoon HH Jin Z Kour O Kankeu Fonkoua LA Shitara K Gibson MK . Association of PD-L1 expression and other variables with benefit from immune checkpoint inhibition in advanced gastroesophageal cancer: systematic review and meta-analysis of 17 phase 3 randomized clinical trials. JAMA Oncol. (2022) 8:1456–65. doi: 10.1001/jamaoncol.2022.3707 PMC9412834 36006624

[80] Zhao Y Ji Z Li J Zhang S Wu C Zhang R . Growth hormone associated with treatment efficacy of immune checkpoint inhibitors in gastric cancer patients. Front Oncol. (2022) 12:917313. doi: 10.3389/fonc.2022.917313 36016614 PMC9395680

[81] Wu W Liu Y Zeng S Han Y Shen H . Intratumor heterogeneity: the hidden barrier to immunotherapy against MSI tumors from the perspective of IFN-γ signaling and tumor-infiltrating lymphocytes. J Hematol Oncol. (2021) 14:160. doi: 10.1186/s13045-021-01166-3 34620200 PMC8499512

[82] Liu J Ma J Xing N Ji Z Li J Zhang S . Interferon-γ predicts the treatment efficiency of immune checkpoint inhibitors in cancer patients. J Cancer Res Clin Oncol. (2023) 149:3043–50. doi: 10.1007/s00432-022-04201-z PMC11796581 35852620

[83] Gholami H Chmiel JA Burton JP Maleki Vareki S . The role of microbiota-derived vitamins in immune homeostasis and enhancing cancer immunotherapy. Cancers (Basel). (2023) 15:1300. doi: 10.3390/cancers15041300 36831641 PMC9954268

[84] Zhang L Kuang T Chai D Deng W Wang P Wang W . The use of antibiotics during immune checkpoint inhibitor treatment is associated with lower survival in advanced esophagogastric cancer. Int Immunopharmacol. (2023) 119:110200. doi: 10.1016/j.intimp.2023.110200 37099942

[85] Hsu PN Yang TC Kao JT Cheng KS Lee YJ Wang YM . Increased PD-1 and decreased CD28 expression in chronic hepatitis B patients with advanced hepatocellular carcinoma. Liver International: Off J Int Assoc Study Liver. (2010) 30:1379–86. doi: 10.1111/j.1478-3231.2010.02323.x 20738778

[86] Gao Q Zhu H Dong L Shi W Chen R Song Z . Integrated proteogenomic characterization of HBV-related hepatocellular carcinoma. Cell. (2019) 179:1240. doi: 10.1016/j.cell.2019.10.038 31730861

[87] Liu B Grindrod N Meyers BM Freiburger S Boldt G Malik A . Treatment modalities to manage hepatocellular carcinoma patients with portal vein thrombosis: a systematic review and meta-analysis. Ann Palliative Med. (2023) 12:1165–74. doi: 10.21037/apm-23-463 37953217

[88] Balermpas P Martin D Wieland U Rave-Fränk M Strebhardt K Rödel C . Human papilloma virus load and PD-1/PD-L1, CD8(+) and FOXP3 in anal cancer patients treated with chemoradiotherapy: Rationale for immunotherapy. Oncoimmunology. (2017) 6:e1288331. doi: 10.1080/2162402x.2017.1288331 28405521 PMC5384387

[89] Chen C Zhang F Zhou N Gu YM Zhang YT He YD . Efficacy and safety of immune checkpoint inhibitors in advanced gastric or gastroesophageal junction cancer: a systematic review and meta-analysis. Oncoimmunology. (2019) 8:e1581547. doi: 10.1080/2162402x.2019.1581547 31069144 PMC6492970

[90] YongCong Y Kai W Kai M ZhiYu X Jie W . Pathogenesis of hepatitis B virus-related hepatocellular carcinoma. J Clin Hepatol. (2020) 36:2167–72.

[91] Zhou L Yang Y Tian D Wang Y . Oxidative stress-induced 1, N6-ethenodeoxyadenosine adduct formation contributes to hepatocarcinogenesis. Oncol Rep. (2013) 29:875–84. doi: 10.3892/or.2013.2227 PMC3597589 23292006

[92] Yang Z Li J Feng G Wang Y Yang G Liu Y . Hepatitis B virus X protein enhances hepatocarcinogenesis by depressing the targeting of NUSAP1 mRNA by miR-18b. Cancer Biol Med. (2019) 16:276–87. doi: 10.20892/j.issn.2095-3941.2018.0283 PMC6713641 31516748

[93] Hu Z Huang P Yan Y Zhou Z Wang J Wu G . Hepatitis B virus X protein related lncRNA WEE2-AS1 promotes hepatocellular carcinoma proliferation and invasion. Biochem Biophys Res Commun. (2019) 508:79–86. doi: 10.1016/j.bbrc.2018.11.091 30471857

[94] Lan T Chang L Wu L Yuan YF . IL-6 plays a crucial role in HBV infection. J Clin Trans Hepatol. (2015) 3:271–6. doi: 10.14218/jcth.2015.00024 PMC4721895 26807383

[95] Wang ZY Tao QF Wang ZH Lin KY Huang G Yang Y . Antiviral therapy improves post-operative survival outcomes in patients with HBV-related hepatocellular carcinoma of less than 3 cm - A retrospective cohort study. Am J Surgery. (2020) 219:717–25. doi: 10.1016/j.amjsurg.2019.05.016 31266631

[96] Zhang YQ Guo JS . Antiviral therapies for hepatitis B virus-related hepatocellular carcinoma. World J Gastroenterol. (2015) 21:3860–6. doi: 10.3748/wjg.v21.i13.3860 PMC4385532 25852270

[97] Zhang C Xie Y Lai R Wu J Guo Z . Nonsynonymous C1653T mutation of hepatitis B virus X gene enhances Malignancy of hepatocellular carcinoma cells. J Hepatocellular Carcinoma. (2022) 9:367–77. doi: 10.2147/jhc.S348690 PMC9078866 35535232

[98] Zhao B Qiao H Zhao Y Gao Z Wang W Cui Y . HBV precore G1896A mutation promotes growth of hepatocellular carcinoma cells by activating ERK/MAPK pathway. Virol Sinica. (2023) 38:680–9. doi: 10.1016/j.virs.2023.06.004 PMC10590694 37331658

