# Supplementary material for: The load of hepatitis B virus reduces the immune checkpoint inhibitors efficiency in hepatocellular carcinoma patients
Source: Front Immunol. 2024 Nov 27;15:1480520. doi: 10.3389/fimmu.2024.1480520 (PMC11632129; doi:10.3389/fimmu.2024.1480520)
Supplement: Supplementary file 1 [file Table1.docx]

**Table.S1.**Quality assessment: The 55 cohort studies were assessed by the Newcastle Ottawa scale. Abbreviations: NOS, Newcastle Ottawa scale.

| **Authors** | **Selection** | **Comparability** | **Outcome** | **NOS score** |
| --- | --- | --- | --- | --- |
| Kennedy Yao Yi Ng et al. | 4 | 2 | 2 | 8 |
| Guosheng Yuan et al. | 4 | 1 | 2 | 7 |
| Haonan Liu et al. | 4 | 1 | 2 | 7 |
| Mengchao An et al. | 4 | 1 | 3 | 8 |
| Xuqi Sun et al. | 4 | 2 | 2 | 8 |
| Guosheng Yuan, et al. | 4 | 2 | 1 | 7 |
| Rohini Sharma et al. | 4 | 1 | 2 | 7 |
| Yanjun Shen et al. | 4 | 1 | 3 | 8 |
| Shuguang Ju et al. | 4 | 1 | 3 | 8 |
| Pei-Chang Lee et al. | 4 | 1 | 2 | 7 |
| Petros Fessas et al. | 4 | 2 | 2 | 8 |
| Song Chen et al. | 4 | 1 | 3 | 8 |
| Junlin Yao et al. | 4 | 2 | 2 | 8 |
| Fucun Xie et al. | 4 | 2 | 2 | 8 |
| Francisca-­Dora Copil et al. | 4 | 2 | 3 | 9 |
| Jaekyung Cheon et al. | 4 | 2 | 1 | 7 |
| Yujing Xin et al. | 4 | 1 | 3 | 8 |
| Lorenz Balcar et al. | 4 | 1 | 3 | 8 |
| Jing Li et al. | 4 | 0 | 3 | 7 |
| Huttakan Navadurong et al. | 4 | 0 | 3 | 7 |
| Mathew Vithayathil et al. | 4 | 1 | 2 | 7 |
| Claudia Campani et al. | 4 | 0 | 3 | 7 |
| Yue Linda Wu et al. | 4 | 1 | 3 | 8 |
| Dongbo CHEN et al. | 4 | 0 | 3 | 7 |
| De-Zhen Guo et al. | 4 | 1 | 3 | 8 |
| Xindan Kang et al. | 4 | 0 | 3 | 7 |
| Qingyan Liu et al. | 4 | 0 | 3 | 7 |
| Xinhua Zou et al. | 4 | 0 | 2 | 6 |
| Xu Chang et al. | 4 | 1 | 2 | 7 |
| Zhongjing Huang et al. | 4 | 1 | 2 | 7 |
| Fei Cao et al. | 4 | 0 | 3 | 7 |
| Huilan Zeng et al. | 4 | 0 | 3 | 7 |
| Kang Wang et al. | 4 | 1 | 2 | 7 |
| Xiaoyun Hu et al. | 4 | 0 | 2 | 6 |
| Lu‑shan Xiao et al. | 4 | 1 | 2 | 7 |
| Jia-Ren Wang et al. | 4 | 1 | 3 | 8 |
| Yusheng Guo et al. | 4 | 1 | 3 | 8 |
| Bang-Bin Chen et al. | 4 | 1 | 3 | 8 |
| Haonan Liu et al. | 4 | 1 | 3 | 8 |
| Bai-Bei Li et al. | 4 | 1 | 2 | 7 |
| Lei Xu et al. | 4 | 1 | 2 | 7 |
| Baizhu Xiong et al. | 4 | 1 | 2 | 7 |
| Jiajia Du et al. | 4 | 1 | 3 | 8 |
| Wei-Fan Hsu et al. | 4 | 1 | 2 | 7 |
| Lu-­Shan Xiao et al. | 4 | 1 | 2 | 7 |
| Yue Chen et al. | 4 | 1 | 2 | 7 |
| Philippe Sultanik et al. | 4 | 1 | 3 | 8 |
| Jiaxin Han et al. | 4 | 1 | 2 | 7 |
| Di Pan et al. | 4 | 2 | 2 | 8 |
| Darren Cowzer et al. | 4 | 0 | 3 | 7 |
| Jiao Zhang et al. | 4 | 1 | 2 | 7 |
| Kun-Peng Ma, et al. | 4 | 1 | 2 | 7 |
| Bang-Bin Chen et al. | 4 | 1 | 3 | 8 |
| WenChi Wu et al. | 4 | 1 | 2 | 7 |
| Michael S Lee et al. | 4 | 1 | 3 | 8 |

**Figure.S1.**The tree diagram for subgroup analysis of HBV versus non-HBV. A: pooled HR of OS on the combination of ICIs with targeted therapy; B: pooled HR of OS on the ICIs monotherapy; C: pooled HR of PFS on the combination of ICIs with targeted therapy; D: pooled HR of PFS on the ICIs monotherapy.


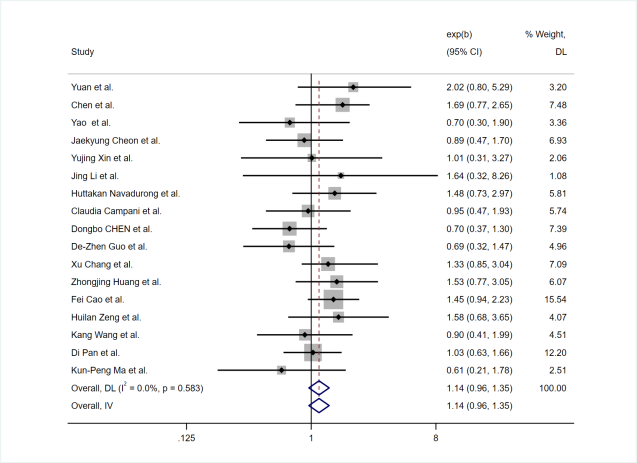


A


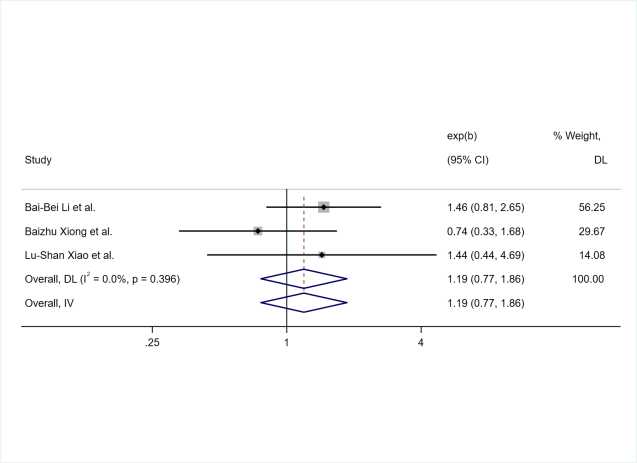


B


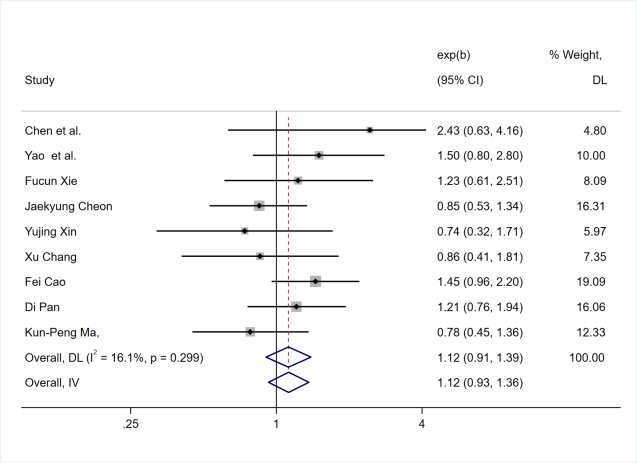


C


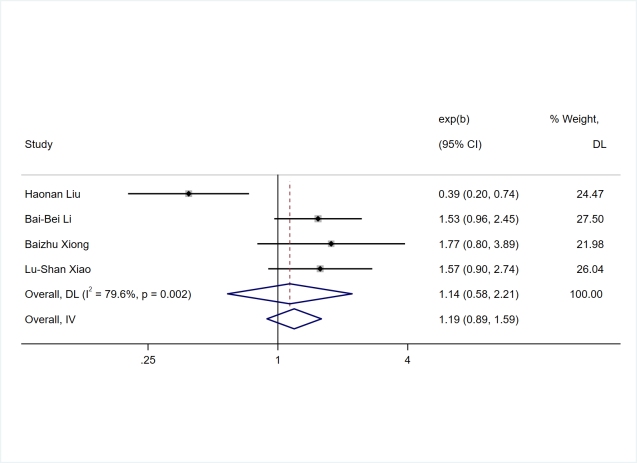


D

**Figure.S2.**The tree diagram for alcohol-induced HCC, NASH-induced HCC and cirrhosis.A: pooled HR of OS on alcohol vs. non-alcohol; B: pooled HR of PFS on alcohol vs. Non-alcohol; C: pooled HR of OS on NASH vs. non-NASH; D: pooled HR of OS on cirrhosis vs. Non-cirrhosis; E: pooled HR of PFS on cirrhosis vs. Non-cirrhosis.


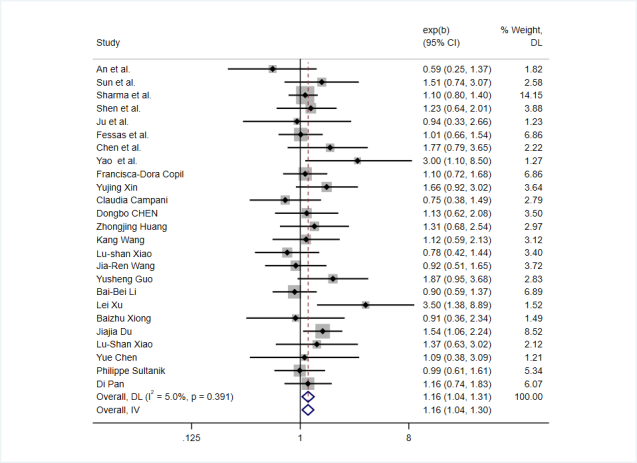


D


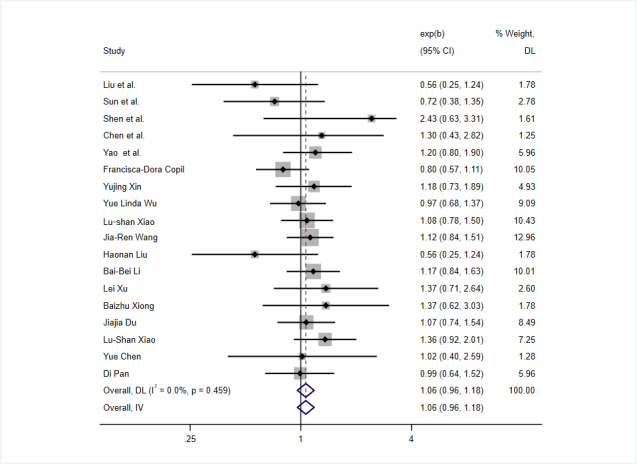


E


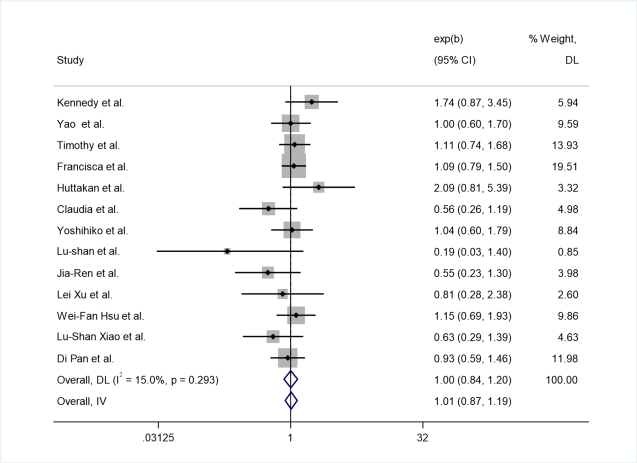


A


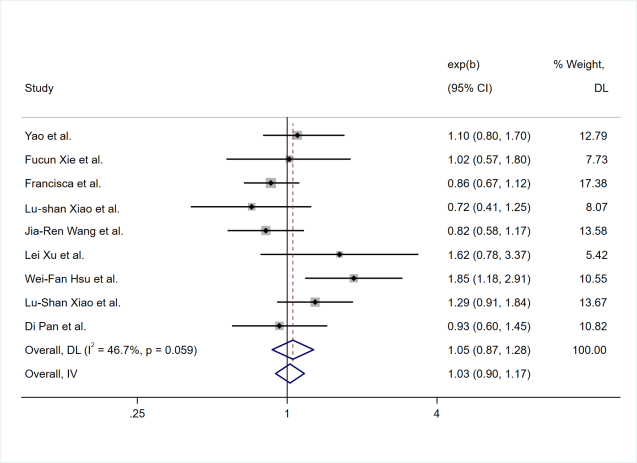


B


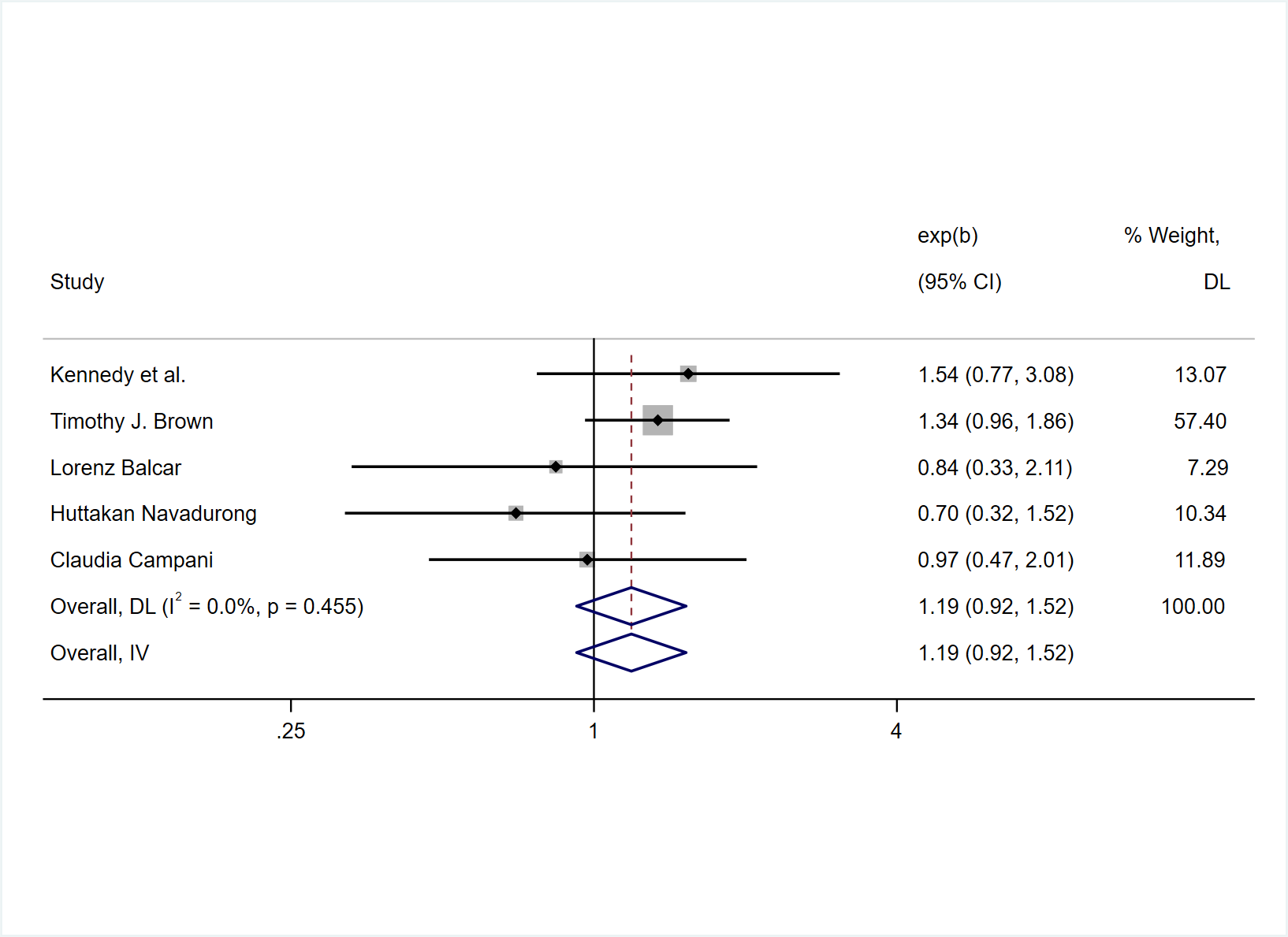


C

**Figure.S3** Sensitivity analysis diagram. A: 35 cohort studies that provided HRs for OS on HBV infection vs. Non-HBV infecction. B: 25 cohort studies that provided HRs for PFS on HBV infection vs. Non-HBV infecction. C: 26 cohort studies that provided HRs for OS on cirrhosis vs. Non-cirrhosis. D: 18 cohort studies that provided HRs for PFS on cirrhosis vs. Non-cirrhosis.

A

B

D

C

**Figure.S4.** Funnel plots. A:26 cohort studies that provided HRs for OS on cirrhosis vs. Non-cirrhosis. B: 18 cohort studies that provided HRs for PFS on cirrhosis vs. Non-cirrhosis.


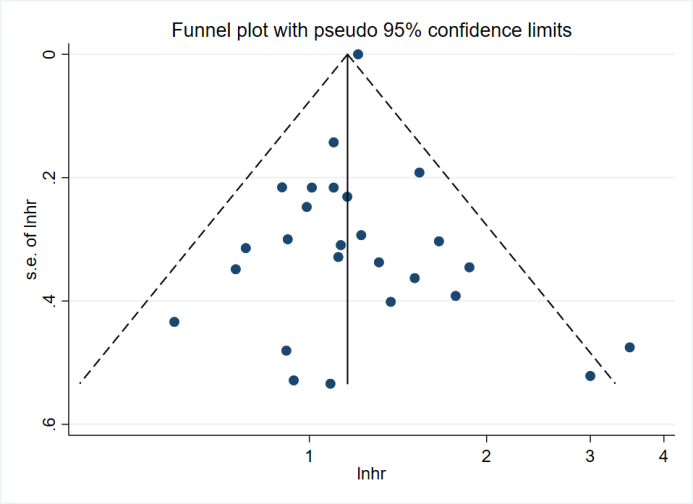


A


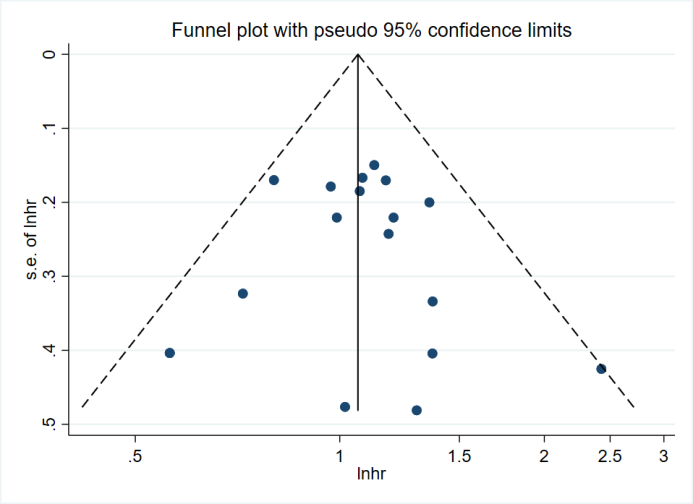


B

**Table.S2.** Symmetry test of funnel plots. A: 35 cohort studies that provided HRs for OS on HBV infection vs. Non-HBV infecction. B: 25 cohort studies that provided HRs for PFS on HBV infection vs. Non-HBV infecction. C: 26 cohort studies that provided HRs for OS on cirrhosis vs. Non-cirrhosis. D: 18 cohort studies that provided HRs for PFS on cirrhosis vs. Non-cirrhosis.

|  | A | B | C | D |
| --- | --- | --- | --- | --- |
| Begg’s test | 0.932 | 0.441 | 0.327 | 0.705 |
| Egger’s test | 0.807 | 0.773 | 0.331 | 0.996 |
